# Supplementary material for: Quantum computational study of chloride attack on chloromethane for chemical accuracy and quantum noise effects with UCCSD and k-UpCCGSD ansatzes
Source: Sci Rep. 2022 May 6;12:7495. doi: 10.1038/s41598-022-11537-6 (PMC9076662; doi:10.1038/s41598-022-11537-6)
Supplement: Supplementary file 1 — Supplementary Information. [file 41598_2022_11537_MOESM1_ESM.pdf]

Supporting Information for

Quantum computational study of chloride attack on chloromethane for chemical accuracy and quantum noise effects with UCCSD and k-UpCCGSD ansatzes

Hocheol Lim[a,b,c], Hyeon-Nae Jeon[c], June-Koo Rhee[d], Byungdu Oh[e,f,*], and Kyoung Tai No[a,b,e,*]

[a] The Interdisciplinary Graduate Program in Integrative Biotechnology and Translational Medicine, Yonsei University, Incheon, Republic of Korea

[b] Bioinformatics and Molecular Design Research Center (BMDRC), Incheon, Republic of Korea

[c] Department of Biotechnology, Yonsei University, Seoul, Republic of Korea

[d] QuNova Computing, Inc. Daejeon, Republic of Korea

[e] Baobab AiBIO Co., Ltd., Incheon, Republic of Korea

[f] SKKU Advanced Institute of Nanotechnology, Sungkyunkwan University, Suwon, Republic of Korea

*Co-corresponding author: Byungdu Oh (bdoh@skku.edu) and Kyoung Tai No (ktno@yonsei.ac.kr)

The supporting information for 'Quantum computational study of chloride attack on chloromethane for chemical accuracy and quantum noise effects with UCCSD and k-UpCCGSD ansatzes' includes Figure S1 for energy diagram of the system of chloride ion attack on chloromethane, Figure S2 for the potential energy surfaces from semi-QM methods, and Figure S3 for the potential energy surfaces from quantum noise simulations with 1,2,3,4-UpCCGSD ansatzes. It includes Table S1 for the RMSE values between UCCSD and k-UpCCGSD ansatzes in quantum noiseless simulation with Qulacs-based arbitrary noise model in this work.

In this study, there are many abbreviations as follows. AM1, Austin model 1; BK, Bravyi-Kitaev; DFT, Density functional theory; DFTB, Density functional tight binding; FCI, Full configurational interaction; FMO, Fragment molecular orbitals; FQE, Full quantum eigensolver; HF, Hartree-Fock; HQA, Hybrid quantum-classical arrangement; JW, Jordan-Wigner; MM, Molecular mechanics; MO, Molecular orbital; NDDO, Neglect of differential diatomic overlap; NISQ, Noisy intermediate scale quantum; PES, Potential energy surface; PM6, Parametric method 6; QM, Quantum mechanics; QPEA, Quantum phase estimation algorithms; RMSE, Root-mean square error; S_N2, Bimolecular nucleophilic substitution; UCC, Unitary coupled cluster; VQA, Variational quantum algorithms; VQE, Variational quantum eigensolver.

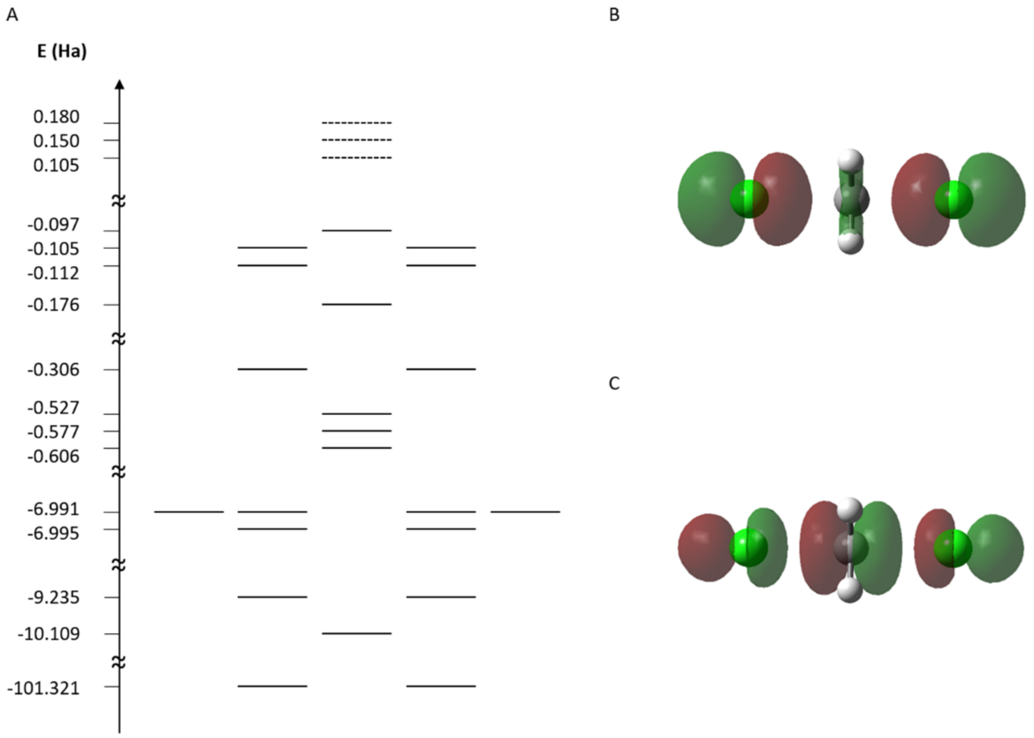

Figure S1. Energy diagram of the system of chloride ion attack on chloromethane.
(A) The energy diagram was generated with 6-31+G*/B3LYP level, which has 22 occupied molecular orbitals (MOs) and 49 virtual MOs. Because the STO-3G level has 22 occupied MOs and 3 virtual MOs, only 25 MOs are shown in this energy diagram. The solid lines correspond to occupied MOs, while the dotted lines represent virtual MOs. (B) The MO of HOMO. (C) The MO of LUMO. The isovalue of MOs is 0.04 and the MO of the positive and negative isovalues are shown in red and green, respectively.

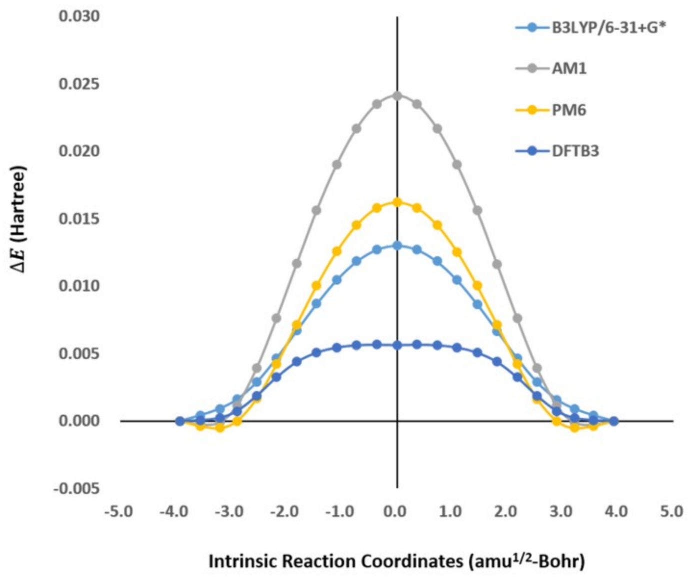

Figure S2. The potential energy surface (PES) from semi-quantum mechanical methods.

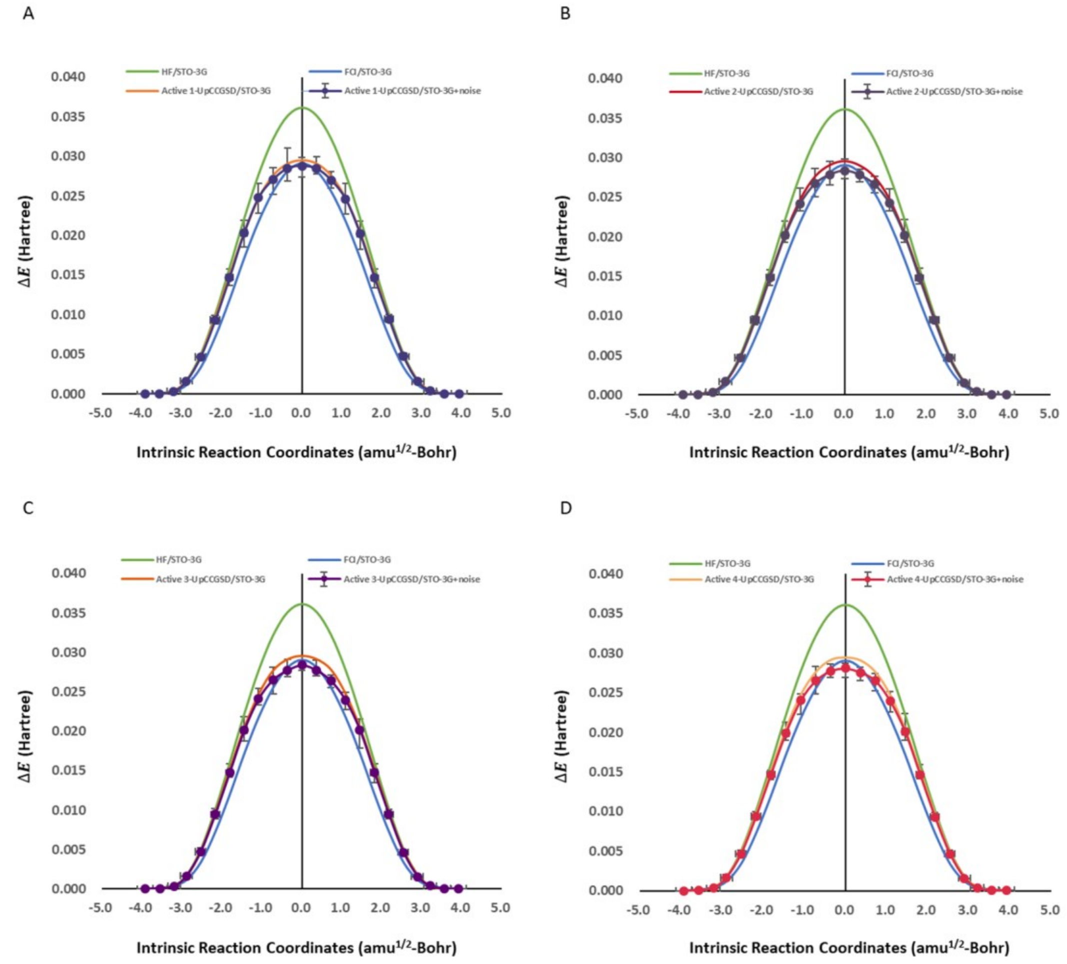

Figure S3. Quantum noisy effects on k-UpCCGSD ansatzes with STO-3G and HOMO/LUMO active space. The classical QM results of HF/STO-3G and FCI/STO-3G without active space are shown in green and blue, respectively. The noiseless results from k-UpCCGSD has performed with STO-3G and HOMO/LUMO active space (*k*=1,2,34). The error bar based from 262,144 shots from 32 times 8,192 shots in quantum noises are shown in black line.

Table S1. The RMSE between UCCSD and k-UpCCGSD in noiseless simulations (kcal/mol)

Active Space Ansatz	Activie Space UCCSD	Active Space 1-UpCCGSD	Active Space 2-UpCCGSD	Active Space 3-UpCCGSD	Active Space 4-UpCCGSD	Active Space 5-UpCCGSD
UCCSD	0.00	2.87×10^{-2}	2.54×10^{-2}	3.17×10^{-2}	1.98×10^{-2}	2.07×10^{-2}
1-UpCCGSD	-	0.00	3.66×10^{-2}	3.91×10^{-2}	2.26×10^{-2}	3.66×10^{-2}
2-UpCCGSD	-	-	0.00	3.99×10^{-2}	3.29×10^{-2}	2.99×10^{-2}
3-UpCCGSD	-	-	-	0.00	2.99×10^{-2}	3.48×10^{-2}
4-UpCCGSD	-	-	-	-	0.00	2.40×10^{-2}
5-UpCCGSD	-	-	-	-	-	0.00

The Qiskit code for 1-UpCCGSD ansatz in this work.

```
from qiskit import QuantumRegister, ClassicalRegister, QuantumCircuit
from numpy import pi

qreg_q = QuantumRegister(4, 'q')
creg_c = ClassicalRegister(4, 'c')
circuit = QuantumCircuit(qreg_q, creg_c)

circuit.u2(1.5707963267948966, -1.5707963267948966, qreg_q[0])
circuit.cx(qreg_q[0], qreg_q[1])
circuit.ry(-1.5707963267948966, qreg_q[2])
circuit.cx(qreg_q[1], qreg_q[2])
circuit.cx(qreg_q[2], qreg_q[3])
circuit.rz(0.0003543554339557886, qreg_q[3])
circuit.cx(qreg_q[2], qreg_q[3])
circuit.cx(qreg_q[1], qreg_q[2])
circuit.cx(qreg_q[0], qreg_q[1])
circuit.u2(1.5707963267948966, -3.141592653589793, qreg_q[0])
circuit.cx(qreg_q[0], qreg_q[1])
circuit.u2(0, 1.5707963267948966, qreg_q[2])
circuit.cx(qreg_q[1], qreg_q[2])
circuit.rz(-0.0003543554339557886, qreg_q[2])
circuit.cx(qreg_q[1], qreg_q[2])
circuit.cx(qreg_q[0], qreg_q[1])
circuit.u2(0, 1.5707963267948966, qreg_q[0])
circuit.u2(1.5707963267948966, -3.141592653589793, qreg_q[2])
circuit.cx(qreg_q[0], qreg_q[2])
circuit.rz(0.0003543554339557886, qreg_q[2])
circuit.cx(qreg_q[0], qreg_q[2])
circuit.u2(1.5707963267948966, -3.141592653589793, qreg_q[0])
circuit.u2(0, 1.5707963267948966, qreg_q[2])
circuit.cx(qreg_q[0], qreg_q[2])
circuit.cx(qreg_q[2], qreg_q[3])
circuit.rz(-0.0003543554339557886, qreg_q[3])
circuit.cx(qreg_q[2], qreg_q[3])
circuit.rz(-0.0003543554339557886, qreg_q[2])
circuit.cx(qreg_q[0], qreg_q[2])
circuit.u2(0, 1.5707963267948966, qreg_q[0])
circuit.u2(1.5707963267948966, -3.141592653589793, qreg_q[2])
circuit.cx(qreg_q[0], qreg_q[2])
circuit.cx(qreg_q[2], qreg_q[3])
circuit.rz(0.0003543554339557886, qreg_q[3])
circuit.cx(qreg_q[2], qreg_q[3])
circuit.cx(qreg_q[0], qreg_q[2])
circuit.u2(1.5707963267948966, -3.141592653589793, qreg_q[0])
circuit.cx(qreg_q[0], qreg_q[1])
circuit.u2(0, 1.5707963267948966, qreg_q[2])
circuit.cx(qreg_q[1], qreg_q[2])
circuit.cx(qreg_q[2], qreg_q[3])
circuit.rz(-0.0003543554339557886, qreg_q[3])
circuit.cx(qreg_q[2], qreg_q[3])
circuit.cx(qreg_q[1], qreg_q[2])
circuit.cx(qreg_q[0], qreg_q[1])
```

```
circuit.u2(0, 1.5707963267948966, qreg_q[0])
circuit.cx(qreg_q[0], qreg_q[1])
circuit.u2(1.5707963267948966, -3.141592653589793, qreg_q[2])
circuit.cx(qreg_q[1], qreg_q[2])
circuit.rz(0.0003543554339557886, qreg_q[2])
circuit.cx(qreg_q[1], qreg_q[2])
circuit.cx(qreg_q[0], qreg_q[1])
circuit.u2(1.5707963267948966, -3.141592653589793, qreg_q[0])
circuit.rx(1.5707963267948966, qreg_q[1])
circuit.cx(qreg_q[0], qreg_q[1])
circuit.cx(qreg_q[1], qreg_q[2])
circuit.rz(0.0036400144454091787, qreg_q[2])
circuit.cx(qreg_q[1], qreg_q[2])
circuit.cx(qreg_q[0], qreg_q[1])
circuit.u2(0, 1.5707963267948966, qreg_q[0])
circuit.cx(qreg_q[0], qreg_q[1])
circuit.u2(0, 1.5707963267948966, qreg_q[2])
circuit.cx(qreg_q[1], qreg_q[2])
circuit.rz(0.0036400144454091787, qreg_q[2])
circuit.cx(qreg_q[1], qreg_q[2])
circuit.cx(qreg_q[0], qreg_q[1])
circuit.rx(-1.5707963267948966, qreg_q[0])
circuit.cx(qreg_q[0], qreg_q[1])
circuit.rx(-1.5707963267948966, qreg_q[2])
circuit.cx(qreg_q[1], qreg_q[2])
circuit.rz(0.0036400144454091787, qreg_q[2])
circuit.cx(qreg_q[1], qreg_q[2])
circuit.cx(qreg_q[0], qreg_q[1])
circuit.u2(-8.881784197001252e-16, -3.141592653589793, qreg_q[0])
circuit.cx(qreg_q[1], qreg_q[3])
circuit.u2(-8.881784197001252e-16, -3.141592653589793, qreg_q[2])
circuit.cx(qreg_q[0], qreg_q[2])
circuit.rz(-0.0036400144454091787, qreg_q[3])
circuit.cx(qreg_q[1], qreg_q[3])
circuit.u2(-1.5707963267948966, -3.141592653589793, qreg_q[1])
circuit.cx(qreg_q[1], qreg_q[2])
circuit.cx(qreg_q[2], qreg_q[1])
circuit.h(qreg_q[1])
circuit.cx(qreg_q[2], qreg_q[0])
circuit.h(qreg_q[0])
circuit.barrier(qreg_q[0], qreg_q[1], qreg_q[2], qreg_q[3])
circuit.measure(qreg_q[0], creg_c[0])
circuit.measure(qreg_q[1], creg_c[1])
circuit.measure(qreg_q[2], creg_c[2])
circuit.measure(qreg_q[3], creg_c[3])
```
